# Ruxolitinib for essential thrombocythemia?

**DOI:** 10.18632/oncoscience.373

**Published:** 2017-11-01

**Authors:** Prithviraj Bose, Srdan Verstovsek

**Affiliations:** Department of Leukemia, UT MD Anderson Cancer Center, Houston, TX, USA

**Keywords:** essential thrombocythemia, ruxolitinib, JAK inhibitor, symptoms, platelet count, leukocyte count

Essential thrombocythemia (ET) is the most indolent of the classic Philadelphia chromosome negative (Ph-) myeloproliferative neoplasms (MPN), characterized by prolonged survival and low rates of progression to post-ET myelofibrosis (post-ET MF) and leukemic transformation. [1] As such, therapy for patients with ET focuses on the prevention of thrombo-hemorrhagic complications. The revised International Prognostic Score for ET (IPSET) - thrombosis model divides patients with ET into four thrombotic risk categories: very low, low, intermediate and high, based on age, presence or absence of the *JAK2^V617F^* mutation and a history of thrombosis.[2] Cytoreductive therapy is generally recommended for patients at intermediate or high risk,[2] and hydroxyurea (HU) is typically the agent of choice. Anagrelide or interferon is usually chosen as second-line therapy in patients who fail HU, although either agent may also be used upfront. A formal definition of resistance/intolerance to HU in patients with ET exists[3], however, this does not address the frequently encountered issue of persistent symptoms despite adequate control of hematologic parameters.

The Janus kinase 1/2 inhibitor ruxolitinib is currently approved for the treatment of patients with myelofibrosis (MF), as well as for HU-resistant/intolerant patients with polycythemia vera (PV). The results of long-term follow-up of 39 patients with ET refractory to or intolerant of HU who were enrolled in a single-arm, non-comparative phase 2 clinical trial of ruxolitinib were recently published.[4] The median platelet and leukocyte counts and hemoglobin levels decreased over the first 4-8 weeks of therapy and stabilized thereafter. Of 22 patients with baseline platelet counts >400 × 10^9^/L, all of whom received a starting dose of 25 mg twice daily (bid) of ruxolitinib, the numbers achieving platelet counts ≤400 × 10^9^/L were rather modest. However, when considering the 20 patients with baseline platelets >600 × 10^9^/L, the proportions achieving platelet counts ≤600 × 10^9^/L were 55%, 40% and 45% at weeks 12, 48 and 312, respectively. The median leukocyte count fell from 8.15 × 10^9^/L at baseline to 5.2 × 10^9^/L by 4 weeks of treatment, and remained within normal limits thereafter (∼5-8 × 10^9^/L). Most evaluable patients derived significant benefit in terms of ET-related symptoms by week 12 of therapy. The median change in the *JAK2^V617F^* allele burden was only -2.8% at 24 weeks, but the response deepened considerably over time, reaching a median of -60% at week 312. There were two thrombotic and four hemorrhagic events on study among the 22 patients who started ruxolitinib at a dose of 25 mg bid. Two of these patients developed grade ≥3 infections, while three had herpes zoster (all grades 1/2).

The MAJIC trial, the ET arm of which was also recently published[5], was a randomized controlled trial (RCT) that compared ruxolitinib (n = 58) to best available therapy (BAT, n = 52) in 110 ET patients resistant to or intolerant of HU. The study found no significant differences between the two arms in terms of complete hematologic response (CHR) rates, rates of thrombosis, hemorrhage, transformation, treatment discontinuation or switching; however, patients receiving ruxolitinib experienced greater symptom improvement than those assigned to BAT.

Although normalization of the platelet count is the goal of cytoreductive therapy in ET, there is no good evidence that platelet counts at baseline or during follow-up correlate with thrombotic risk; indeed, in one study of 891 patients with World Health Organization-defined ET, a platelet count >1000 × 10^9^/L at baseline was associated with a lower risk of arterial thrombosis.[6] Baseline leukocytosis >11 × 10^9^/L predicted arterial, but not venous, thrombosis in this study.[6] A significant association was reported between abnormal platelet counts during follow-up and an immediate risk of major hemorrhage (but not thrombosis) in an analysis of the prospective PT-1 cohort (n = 776).[7] Leukocytosis during follow-up correlated with the occurrence of both thrombosis and major bleeding in this study.[7] The ongoing US pivotal RCT (see Table 1) of ruxolitinib versus anagrelide in patients with ET resistant to or intolerant of HU requires leukocyte and platelet counts at screening of >11 × 10^9^/L and >650 × 10^9^/L, respectively.

**Table 1:** Ongoing comparative clinical trials evaluating ruxolitinib in adults with essential thrombocythemia

Clinicaltrials.gov identifier	Other name	Phase	Sponsor	Comparator	Major eligibility criteria
NCT03123588	RESET-272	2	Incyte Corporation	Anagrelide	HU resistance or intolerance;[3] platelets >650 × 10^9^/L and leukocytes >11 × 10^9^/L at screening
NCT02962388	RUXBETA	2/3	French Innovative Leukemia Organization	Anagrelide or interferon alfa (can be pegylated)	Patients with high risk ET and HU resistance or intolerance[3]
NCT02577926	Ruxo-BEAT	3	RWTH Aachen University	Best available therapy	Treatment-naïve or previously treated patients who are >60 or have platelets >1500 × 10^9^/L, or h/o prior thromboembolism or ET-related severe hemorrhage

Abbreviations: ET, essential thrombocythemia; HU, hydroxyurea.

Patients with ET have a substantial symptom burden, with 72% reporting fatigue and ≥40% reporting itching, night sweats and bone pain in one large internet-based survey (n = 304).[8] Given that ruxolitinib, highly effective in controlling symptoms in patients with MF and PV through suppression of cytokines, is not currently approved for patients with ET, the treatment of symptomatic patients with ET who fail standard therapies remains an area of unmet need. Unfortunately, neither the ongoing registration trial in the US of ruxolitinib (NCT03123588) nor the second-line trial ongoing in Europe (NCT02962388) permit the enrollment of patients with uncontrolled symptoms on HU, unless conventional criteria for resistance/intolerance[3] are met. The RUXO-BEAT trial (NCT02577926) in Germany comparing ruxolitinib to BAT does allow the enrollment of both treatment-naïve and previously treated patients with ET as long as ≥1 high risk features (age >60, platelets >1500 × 10^9^/L, previous thrombosis or severe hemorrhage related to ET) are present.

Potential concerns surrounding the use of ruxolitinib in patients with an indolent condition like ET include cost of therapy and long-term infectious risk, although the latter is not a major concern based on the experience to date in MF and PV. While driver mutation allele burden reduction over time, also seen in PV, is intuitively appealing, the significance of this is poorly understood, e.g., progression of ET to MF despite attainment of complete molecular response was reported in the MAJIC trial.[5]
